# One-Pot Colorimetric Nucleic Acid Test Mediated by Silver Nanoparticles for DNA Extraction and Detection

**DOI:** 10.3390/bios15050271

**Published:** 2025-04-25

**Authors:** Seung Kyun Park, Kieu The Loan Trinh, Nae Yoon Lee

**Affiliations:** 1 Department of BioNano Technology, Gachon University, 1342 Seongnam-daero, Sujeong-gu, Seongnam-si 13120, Gyeonggi-do, Republic of Korea; dnsl106@gachon.ac.kr; 2 BioNano Applications Research Center, Gachon University, 1342 Seongnam-daero, Sujeong-gu, Seongnam-si 13120, Gyeonggi-do, Republic of Korea; tktloan@gmail.com

**Keywords:** one-pot colorimetric nucleic acid test, polyethyleneimine, silver nanoparticles, glass fiber membrane, loop-mediated isothermal amplification, sodium ascorbate, colorimetric detection

## Abstract

This study introduces a one-pot colorimetric nucleic acid test (NAT) platform that integrates silver nanoparticle (AgNP)-based DNA isolation and colorimetric detection of bacterial genes. The NAT platform is comprised with purification and reaction units that enable cell lysis, DNA purification, loop-mediated isothermal amplification (LAMP), and colorimetric detection. In the purification unit, polyethyleneimine (PEI)-capped AgNPs were used as cell lysis agents because of their cell-disrupting and antimicrobial properties and were immobilized on a glass fiber membrane for DNA capture and isolation. The reaction unit enabled colorimetric detection of DNA amplicons, achieved by the synthesis of AgNPs on chromatography paper formed via the reduction of silver ions present on the paper, mediated by the use of sodium ascorbate, a reducing agent, present in the LAMP reagent, after the reaction. AgNPs were formed only in the presence of the target amplicons in the positive samples after reaction at 65 °C for 5 min. Bacterial DNA was efficiently extracted using this method, and *Enterococcus faecium* was detected with a detection limit of 10^2^ CFU/mL. This platform is a promising alternative for rapid and cost-effective nucleic acid testing in resource-limited settings.

## 1. Introduction

The realms of molecular biology and diagnostics are evolving rapidly, necessitating new techniques for effective nucleic acid testing (NAT) [1,2,3,4]. NAT plays a pivotal role in healthcare by enabling the detection of infectious agents, genetic disorders, and cancer biomarkers, thereby facilitating timely and targeted treatments. However, traditional NAT methods often face challenges related to sensitivity, specificity, speed, and cost, which hinder their widespread application in clinical settings. Recently, various developments have been made in one-pot NAT in combination with DNA extraction and detection [5,6]. For example, Shkodenko et al. introduced a finger-activated microfluidic platform for detecting *Neisseria meningitidis* and herpes simplex virus using hydroxynaphthol blue [5]. Furthermore, Rodriguez-Mateos et al. established a lab-on-a-chip platform based on implacable flotation by surface tension and a colorimetric loop-mediated isothermal amplification (LAMP) assay to extract and detect the genomic DNA of *N. gonorrhoeae* [6]. This NAT can be performed rapidly, thereby increasing the cost-effectiveness of experiments and reducing the risk of sample cross-contamination. However, as most of these systems involve DNA extraction using chemical methods, they may not function perfectly in all cells or tissues, and cell residues, proteins, and other organic substances may be extracted together with DNA, affecting the detection process.

Nanoparticles are ideal for isolating DNA owing to their high surface-to-volume ratio, ability to remain suspended in solution, and customizable surface charges. Silver nanoparticles (AgNPs) are particularly advantageous because they are cost-effective, require less processing time for synthesis, and can be used without extensive surface modifications [7]. Moreover, AgNPs may be used for the colorimetric detection of various biomolecules, such as dopamine, proteins, and DNA [8]. Their unique optical and antimicrobial properties arise from their small size, which typically ranges from 1 to 100 nm. In addition, the size of AgNPs is not the sole determining factor for antimicrobial efficacy; factors such as shape, surface modification, and concentration also play crucial roles [9]. Numerous studies have demonstrated antibacterial activity against both Gram-positive and Gram-negative bacteria [10,11,12]. AgNPs can aggregate on bacterial cell walls and membranes, leading to significant morphological alterations such as cytoplasmic shrinkage, membrane detachment, the formation of electron-dense pits, and ultimately membrane disruption [13]. They can bind to membrane proteins involved in processes such as electron and molecular transport, thereby significantly affecting membrane permeability, cell division, and ion transport. Upon attachment to cell membranes, AgNPs penetrate bacterial cells and interact with essential cellular structures and biomolecules, including lipids and proteins, thereby inhibiting protein synthesis and disrupting cellular functions. In one study, AgNPs were used to lyse microbial cells for genomic DNA extraction [14]. Polymer coatings and surface modifications of nanoparticles are vital for effective DNA extraction [15]. Cationic polymers are particularly useful for purifying DNA from complex biological mixtures because they attract DNA molecules via electrostatic interactions [16]. Among these, polyethyleneimine (PEI) stands out because of its strong electrostatic interactions with DNA via its primary, secondary, and tertiary amine groups [17]. PEI performs dual functions in the synthesis of metal nanoparticles, acting both as a stabilizer and a reducing agent. The nitrogen atoms in the amine groups can donate electron pairs to interact with AgNPs. Hence, PEI has been used to enhance the antimicrobial efficacy of AgNPs in various contexts [18].

Recent studies have highlighted the application of AgNPs in colorimetric assays for DNA amplicons [19,20]. DNA can act as a counterion, engaging in non-covalent interactions with metal ions to neutralize the negative charges of its phosphate backbone [21]. Consequently, the nitrogenous bases in the DNA reduce Ag ions to form DNA-AgNP conjugates, resulting in a color change from colorless to deep yellow.

In this study, we aimed to improve the stability and biocompatibility of AgNPs by capping them with PEI. Additionally, the immobilization of PEI-capped AgNPs (PEI-AgNPs) on paper surfaces created a practical platform for cell lysis and DNA purification, leveraging the strong interactions between PEI and cellular components. DNA amplification was efficiently conducted at a constant temperature using LAMP. Sodium ascorbate was used as the reducing agent for AgNP synthesis [22]. After amplification in a LAMP mixture containing sodium ascorbate, the resulting color change was verified in a reaction with paper soaked in Ag^+^ ions. This approach allows the detection of LAMP amplicons without pipetting, paving the way for rapid and accessible diagnostic applications.

## 2. Materials and Methods

### 2.1. Materials

Silver nitrate (AgNO_3_) and sodium borohydride (NaBH_4_) were purchased from Sigma-Aldrich (Saint Louis, MO, USA). Branched polyethylenimine (PEI, molecular weight 600) was acquired from Alfa Aesar (Haverhill, MA, USA). Sodium L-ascorbate (C_6_H_7_NaO_6_) was obtained from Tokyo Chemical Industry (Tokyo, Japan). The LAMP kit, which included *Bst* DNA polymerase (2.0), 10× isothermal amplification buffer, and 100 mM magnesium sulfate (MgSO_4_), was purchased from New England BioLabs *(*Ipswich, MA, USA). Agarose powder and the dNTP mix were purchased from BioFACT (Daejeon, South Korea). A 100 bp DNA ladder was obtained from Takara (Shiga, Japan). Ethidium bromide-based DNA intercalation dye (Loading STAR) was purchased from DyneBio (Seongnam, Republic of Korea). The glass fiber (GF) membrane (GF5 grade, 1.2 µm pore size) and chromatography paper (CHR) were purchased from Thermo Fisher Scientific (San Francisco, CA, USA).

### 2.2. Preparation of Bacterial Samples

*Enterococcus faecium* (*E. faecium*, ATCC BAA-2127) was cultured in 5 mL of brain heart infusion (BHI) media and incubated at 37 °C for 16 h, with constant shaking at 200 rpm in a shaking incubator. For generating the DNA template of *Listeria monocytogenes* (*L. monocytogenes*), a partial sequence of the *hly*A gene was synthesized and cloned into a plasmid by Cosmo Genetech (Seoul, Republic of Korea).

### 2.3. Synthesis and Characterization of PEI-AgNPs

**Figure 1:**
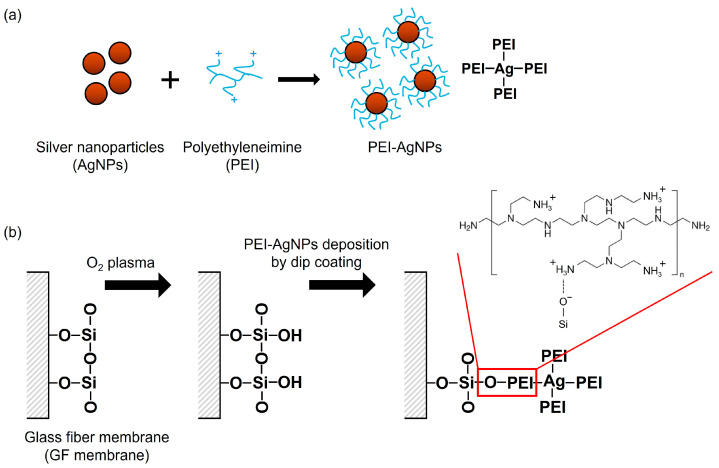
Schematic showing (**a**) synthesis of PEI-AgNPs and (**b**) modification of GF membrane for the coating of PEI-AgNPs.

PEI-AgNPs were synthesized using a slightly modified version of a previously published methodology (Figure 1a) [23]. Briefly, a 0.6% PEI solution was prepared by dissolving PEI in boiling distilled water. AgNO_3_ (10 mM) and 20 mM NaBH_4_ were prepared separately, and 1 mL of PEI solution was added dropwise into 15 mL of AgNO_3_ solution under stirring for 2 h. Afterward, 100 µL of NaBH_4_ solution was added, and the mixture was further stirred at room temperature for 24 h. The absorbance of the synthesized PEI-AgNPs was measured using an Epoch™ microplate spectrophotometer (BioTek Instruments, Inc., Winooski, VT, USA). The morphology of the synthesized PEI-AgNPs was characterized using transmission electron microscopy (TEM).

### 2.4. Live/Dead Bacterial Viability Assay

A live/dead BacLight bacterial viability kit (Invitrogen, Carlsbad, CA, USA) was used to evaluate the effect of PEI-AgNPs on bacterial cell wall disruption. Bacteria were prepared as described previously. Three different samples were prepared. For each sample, 1 mL of the bacterial culture (10^8^ CFU/mL) was centrifuged at 5000× *g* for 10 min, and the supernatant was discarded. For one sample, the pellet was resuspended in 1 mL of 0.85% NaCl as a control. In another sample, the pellet was resuspended in 1 mL of 0.6% PEI. In the other sample, the pellet was resuspended in 1 mL of PEI-AgNPs. After incubating the three samples independently at 65 °C for 10 min, they were centrifuged at 5000× *g* for 10 min, and the supernatants were removed. To thoroughly wash away the remaining PEI and PEI-AgNPs from the solutions, all three samples were resuspended in 1 mL of 0.85% NaCl and centrifuged again at 5000× *g* for 10 min, following which the supernatants were removed. Subsequently, 1 mL of a 0.85% NaCl solution was added to all three samples. Next, 3 µL of the mixture of SYTO 9 and propidium iodide (PI) was added to each sample and incubated at room temperature in the dark for 15 min. Next, 5 µL of the bacterial samples was taken for microscopic observation using an Olympus IX71 fluorescence microscope (Olympus Corporation, Tokyo, Japan). ImageJ software 1.54g was used to quantify cell viability. The proportion of live and dead cells was calculated using the following formula, where the fluorescence intensities of SYTO 9 and PI were measured across specific wavelength ranges to determine the total intensity of each dye [24].

$$\%livecells\propto \frac{SYTO9}{SYTO9+PI}\times 100$$

$$\%deadcells\propto \frac{PI}{SYTO9+PI}\times 100$$

### 2.5. Extraction of Genomic DNA

GF membrane discs (3 mm in diameter), composed of silica (SiO_2_), were prepared by punching the membranes using a manual puncher. In the PEI-AgNP modification process (Figure 1b), the GF discs were initially treated with O_2_ plasma for 1 min to activate the surface. The treated discs were then immersed in the PEI-AgNP solution at room temperature for 24 h. Subsequently, the GF discs were washed thrice with distilled water and air-dried for 10 min.

To isolate genomic DNA, the PEI-AgNP-coated disc was immersed in 0.1 mL of bacterial cell culture solution and was incubated at 65 °C for 10 min. Then, to assess the purity and yield of the isolated DNA attached on the PEI-AgNP-coated disc, the disc was immersed in 50 µL of TE buffer (10 mM Tris and 1 mM EDTA, pH 8.0) at room temperature for 5 min to elute the DNA. The eluted DNA was then assessed for quantity and purity using NanoDrop™ 2000 spectrophotometry (Thermo Fisher Scientific, USA). The LAMP products, obtained by direct incorporation of the PEI-AgNP-coated disc with immobilized DNA into the reaction mixture, were analyzed via agarose gel electrophoresis.

### 2.6. LAMP Reaction

For the *esp* gene of *E. faecium* and *hly*A gene of *L. monocytogenes*, LAMP primer sets were designed using PrimerExplorer. The primer sequences are listed in Table 1. The LAMP reaction was conducted at 65 °C for 45 min using a 25 µL mixture of 1 mM dNTP mix, 6 mM MgSO_4_, 10× isothermal amplification buffer, 1.6 µM of each inner primer (FIP and BIP), 0.2 µM of each outer primer (F3 and B3), 0.8 µM of loop primer, and four units of *Bst* 2.0 DNA polymerase. The reaction results were evaluated using agarose gel electrophoresis. The LAMP reaction contained sodium ascorbate, which was added before the reaction, and various concentrations (30–50 mM) of sodium ascorbate were tested to identify the optimal conditions for amplifying the target DNA. The PEI-AgNP-coated disc, to which the isolated DNA was attached, was used for subsequent LAMP reaction.

### 2.7. Colorimetric Detection

Sodium ascorbate added before the LAMP reaction was used to detect the LAMP amplicons because DNA-bound silver ions are reduced by sodium ascorbate in the presence of DNA and can be detected by the naked eye. Various concentrations of AgNO_3_ (7.5–15 mM) were evaluated to determine the optimum conditions for detecting the LAMP amplicons. The results were assessed by measuring the absorbance via UV–Vis spectrophotometry. Furthermore, the morphology of the AgNPs was analyzed using TEM. To avoid interference from AgNPs, DNA used for colorimetric detection was isolated from the PEI-AgNP-coated GF membrane, ensuring that residual AgNPs were removed during the extraction process.

### 2.8. Fabrication and Operation of the One-Pot Colorimetric NAT Platform

**Figure 2:**
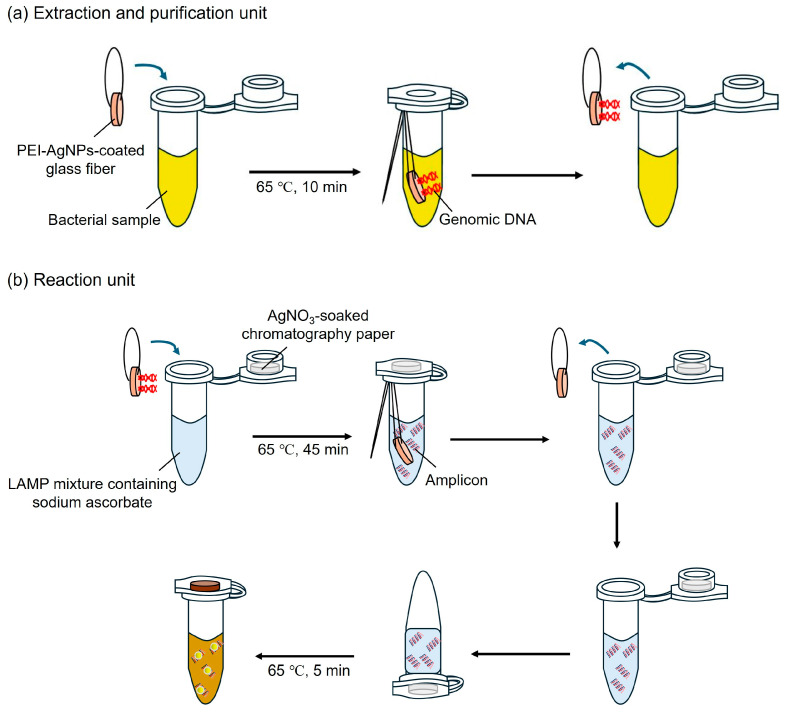
Schematic showing the fabrication and operation of the one-pot colorimetric NAT platform. (**a**) Extraction and purification unit for DNA isolation. (**b**) Reaction unit for LAMP and colorimetric assay.

Figure 2 illustrates the specific layout of the one-pot colorimetric NAT platform, which is composed of two parts: an extraction and purification unit and a reaction unit. The extraction and purification unit comprised a PEI-AgNP-coated GF membrane (3 mm in diameter) and a sample tube. The PEI-AgNP-coated GF membrane was suspended in a tube using nylon threads. The reaction unit consisted of AgNO_3_-soaked chromatography (CHR) paper (4 mm in diameter) and a LAMP mixture tube. An AgNO_3_-soaked CHR disc was inserted into the cap of the LAMP mixture tube.

The platform enables cell lysis, DNA extraction/purification, LAMP reactions, and colorimetric detection. First, 100 µL of the bacterial sample was dropped onto the PEI-AgNP-coated GF disc and heated at 65 °C for 10 min to facilitate cell lysis and DNA extraction/purification. Subsequently, the coated GF disc was removed from the sample tube and added to the LAMP mixture tube to elute DNA from the disc. The LAMP mixture tube was heated at 65 °C for 45 min for the reaction. After the LAMP reaction, the coated GF discs were removed from the tubes. After closing the cap, the tube was turned over to react with the CHR disc inserted into the inner cavity of the inner side of the cap and heated at 65 °C for 5 min to speed up the reaction. Following heating, the tube was inverted back to the upright position, and the presence of LAMP amplicons was confirmed by a change in the solution color from colorless to dark yellow.

## 3. Results and Discussion

### 3.1. Synthesis and Characterization of PEI-AgNPs

**Figure 3:**
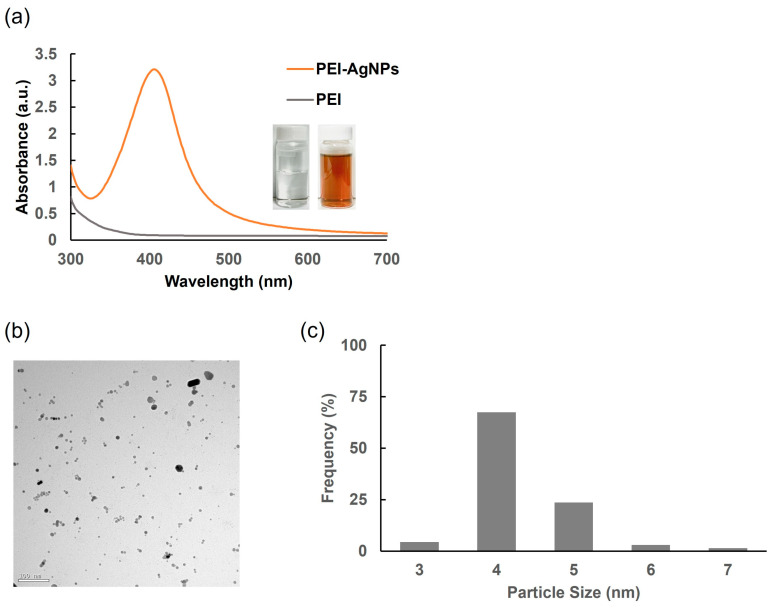
Characterization of chemically synthesized PEI-AgNPs. (**a**) Absorbance of PEI and PEI-AgNPs. (**b**) TEM image of PEI-AgNPs. (**c**) Size distribution of PEI-AgNPs.

The characteristic orange-brown color observed using PEI as a stabilizing agent indicated the successful formation of the AgNPs. The amine groups within the PEI structure act as ligands in the nanoparticle synthesis process, where the nitrogen atoms donate electrons to silver ions, thereby facilitating the reduction and stabilization of the nanoparticles [25]. The UV–Vis absorption spectrum of the PEI-stabilized AgNPs revealed a prominent peak at 406 nm (Figure 3a), which fell within the typical absorption range (400–450 nm) of AgNPs [26]. The specific peak at 406 nm suggested that the nanoparticles were relatively small and well dispersed in the solution. TEM was used to examine the morphology and size distribution of the AgNPs. The TEM micrographs (Figure 3b,c) showed predominantly spherical AgNPs with sizes ranging from approximately 4 to 5 nm. This small size range is consistent with the observed UV–Vis absorption peak, indicating that the PEI stabilization method effectively produces uniformly sized nanoparticles. These results confirmed that PEI is an effective stabilizing agent for the synthesis of AgNPs, enabling stabilization and size control. The observed characteristics of the AgNPs, including their size and optical properties, agreed with the expected outcomes based on the stabilization mechanism of PEI and its interaction with silver ions.

### 3.2. Effect of PEI-AgNPs on Cell Lysis

**Figure 4:**
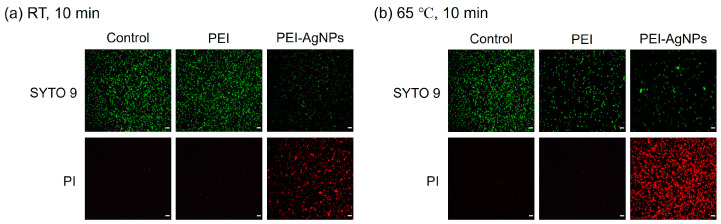
Fluorescence microscopic images of live and dead bacteria after incubating for 10 min at (**a**) room temperature and (**b**) 65 °C. SYTO 9 fluorescence is captured under a blue light excitation wavelength of 485 nm, and the fluorescence of PI is captured under a green light excitation wavelength of 535 nm. The scale bar is 10 µm. Control: untreated bacteria. PEI: bacteria treated with 0.6% PEI. PEI-AgNPs: bacteria treated with PEI-AgNPs.

Figure 4 illustrates the distribution of live and dead bacteria as visualized using fluorescence microscopy. Live bacteria, indicated by green fluorescence, were predominantly observed in the untreated control group, confirming the viability of the control bacteria. To assess the effects of PEI and PEI-AgNP treatment on bacterial cell integrity, two nucleic acid-binding stains, SYTO 9 and PI, were used. SYTO 9 stains all bacterial cells green by penetrating intact membranes, whereas PI stains only those with compromised membranes, producing red fluorescence [27]. As shown in Figure 4a, after incubation at room temperature for 10 min, PEI-treated *E. faecium* predominantly exhibits green fluorescence, indicating that most of the cells remain intact. In contrast, cells treated with PEI-AgNPs show a higher proportion of red fluorescence than green fluorescence, indicating that PEI-AgNP treatment significantly damages the bacterial cell membrane and cell wall, leading to increased permeability and eventual cell death. Figure 4b, which presents the results after incubation at 65 °C for 10 min, shows a similar pattern, with PEI-AgNP-treated cells demonstrating a higher level of red fluorescence than the control and PEI-only treated groups, suggesting enhanced cell membrane disruption at elevated temperatures.

**Figure 5:**
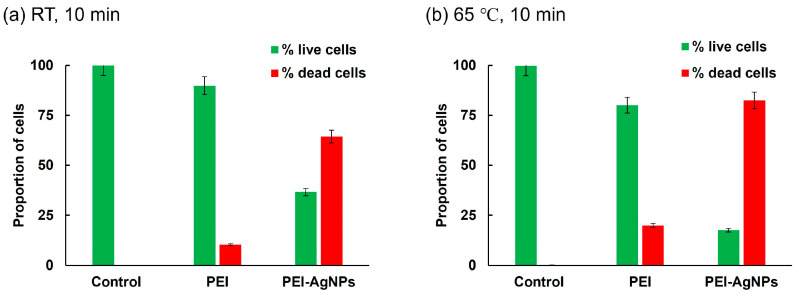
Proportion of live and dead cells. Bacteria treated for 10 min at (**a**) room temperature and (**b**) 65 °C. Experiments were performed using 10^8^ CFU/mL of bacterial cell culture solution.

Figure 5 presents a quantitative analysis of the live and dead cells derived from the fluorescence microscopy images of Figure 4. After incubation at room temperature for 10 min (Figure 5a), the untreated control cells showed minimal cell death (0.1%). Conversely, the PEI-treated cells showed a notable increase in cell death, with 10.2% of the cells exhibiting red fluorescence. This indicates that PEI treatment caused moderate damage to the bacterial cells. However, PEI-AgNP treatment substantially increased cell death, with 63.4% of the cells displaying red fluorescence. This dramatic increase suggests that PEI-AgNPs are significantly more effective in compromising bacterial cell membranes and walls than PEI alone. To further assess the impact of temperature on cell death, a similar experiment was conducted at 65 °C for 10 min (Figure 5b). The proportion of dead cells in the untreated control group was 0.2%, whereas those of the PEI-treated cells and PEI-AgNP-treated cells were 19.9% and 82.5%, respectively. This indicates that PEI-AgNPs not only damaged bacterial cell membranes but also enhanced cell lysis under heat treatment. The elevated cell death rate following treatment with PEI-AgNPs highlights their potential as effective antimicrobial agents. The combination of thermal treatment and PEI-AgNP application synergistically enhanced damage to bacterial cells, possibly due to increased permeability and subsequent disruption of cellular integrity [28].

### 3.3. DNA Extraction Performance

**Figure 6:**
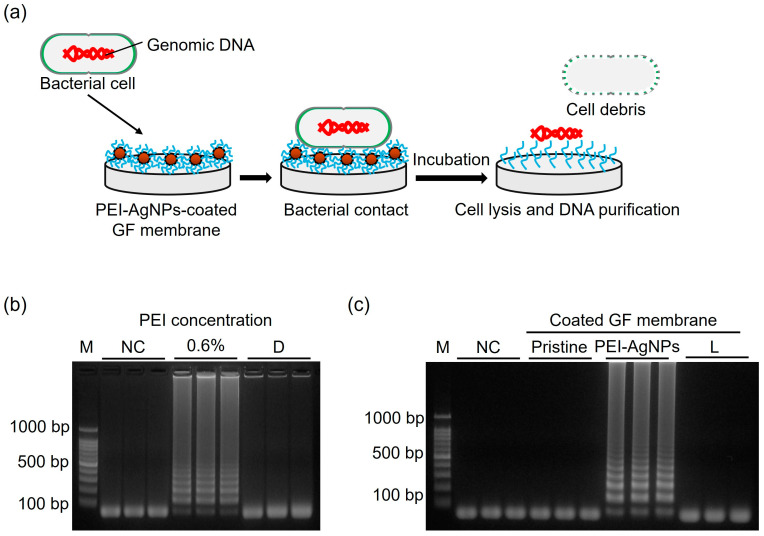
(**a**) Schematic showing the DNA isolation mechanism using immobilized PEI-AgNPs. (**b**) Agarose gel electrophoresis obtained after LAMP reaction when DNA was purified using PEI-AgNP-coated GF membrane. The PEI concentration was 0.6%. (**c**) Agarose gel electrophoresis obtained after LAMP reaction when cell lysis and DNA isolation were performed using PEI-AgNP-coated GF membrane. M: ladder. NC: negative control. D: *E. faecium* heated at 95 °C for 10 min. L: *E. faecium* heated at 65 °C for 10 min.

Figure 6a shows the mechanism of isolating DNA from the immobilized PEI-AgNPs. When bacteria make contact with the PEI-AgNP-coated GF membrane, AgNPs on the coated surface release silver ions, which destroy the bacterial cell membrane [29,30]. Intracellular components such as DNA and ribosomes are released when the cell membrane is destroyed, and the released DNA binds to the positively charged coated surface of PEI, from where it is isolated [31].

Before assessing the efficacy of DNA isolation using PEI-AgNPs, the effect of immobilized 0.6% PEI on DNA purification was investigated. The bacterial sample (100 µL) was heated at 95 °C for 10 min to induce cell lysis. The heated sample was then applied to a PEI-coated GF disc, followed by LAMP (Figure 6b). Successful amplification indicates that heat treatment effectively compromises the bacterial cell membrane and cell wall, leading to the release of intracellular components, including DNA and ribosomes [32]. The interaction between the negatively charged DNA and positively charged PEI facilitates DNA binding, demonstrating the capacity of PEI to capture DNA. PEI becomes electrically neutral when a DNA-bound disc is introduced into an alkaline LAMP mixture, and DNA is released into the solution [33].

To further evaluate the DNA isolation efficiency, we used a PEI-AgNP-coated GF disc, which was immersed in the bacterial sample at 65 °C for 10 min. The temperature was reduced to 65 °C as the extraction was driven by chemicals such as PEI and AgNPs rather than by heat, making the use of high temperatures unnecessary. The subsequent LAMP reaction revealed that the PEI-AgNP-coated GF disc successfully captured DNA while maintaining the cell lysis capability, as evidenced by the detection of DNA target bands in agarose gel electrophoresis (Figure 6c). This suggests that PEI-AgNPs not only enhance DNA binding but also efficiently lyse cells, contributing to effective DNA isolation. To assess the purity and yield of the isolated DNA, the PEI-AgNP-coated disc was then immersed in 50 µL of TE buffer at room temperature for 5 min to elute the DNA. The concentration of the resulting DNA solution was 30.7 ng/µL. The purity of the isolated DNA was evaluated by determining the ratio of the absorbances at 260 nm and 280 nm A ratio of 1.87 indicates the relative abundance of nucleic acids relative to the protein content. A 260:280 ratio of approximately 1.8 to 2.0 is generally considered indicative of high-purity DNA, with values above 1.8 suggesting minimal protein contamination and the presence of high-quality DNA suitable for downstream applications.

### 3.4. Sodium Ascorbate-Induced AgNPs for LAMP Colorimetric Detection

**Figure 7:**
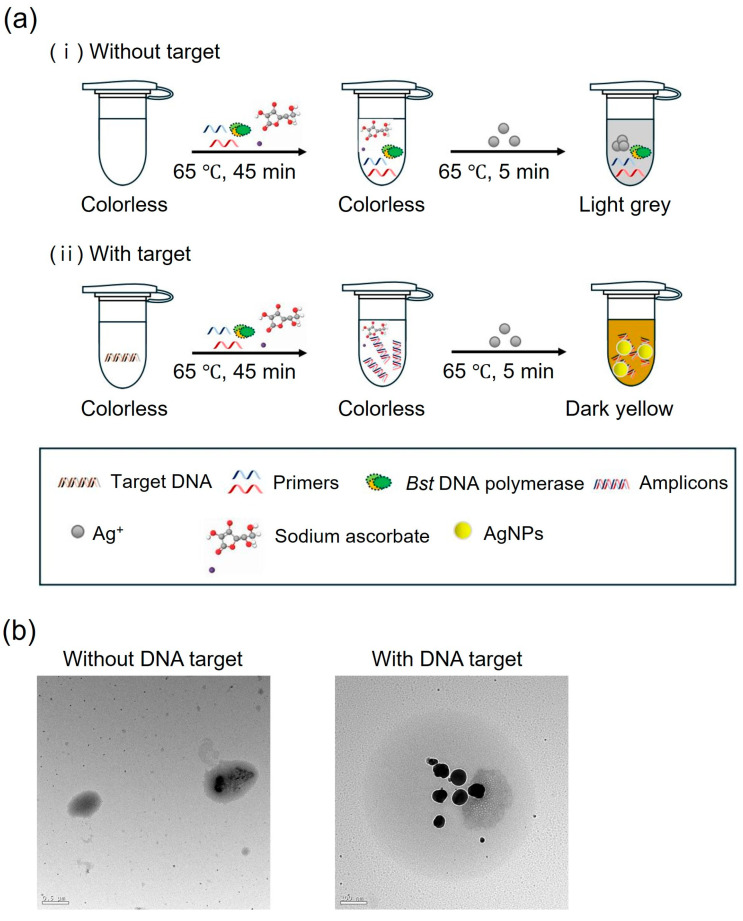
(**a**) Schematic showing colorimetric LAMP detection process in the (**i**) absence and (**ii**) presence of target DNA using silver ions and sodium ascorbate solution. (**b**) TEM images of AgNPs in the absence and presence of the target DNA.

The colorimetric detection method for LAMP uses AgNPs generated in the presence of DNA amplicons. The process begins with the amplification of the target DNA via LAMP, producing numerous DNA amplicons. In this study, sodium ascorbate was introduced into the solution as a reducing agent. Silver ions were then added to the solution, where they bind to the DNA amplicons. Sodium ascorbate facilitates the reduction of silver ions, leading to the formation of AgNPs. In the presence of the target DNA, the solution turns dark yellow, indicating the formation of the AgNPs. In the absence of the target DNA, the solution remains light gray, demonstrating the absence of AgNP formation (Figure 7a). TEM images (Figure 7b) confirmed the presence and morphology of AgNPs. In the absence of the target DNA, the silver ions were oxidized, and nanoparticles were not formed. In contrast, the presence of the target DNA led to the formation and dispersion of AgNPs.

**Figure 8:**
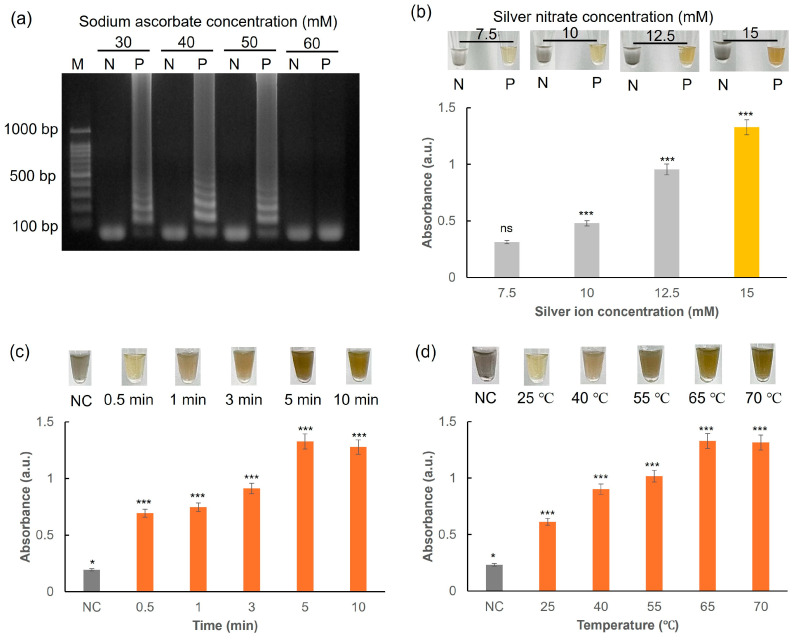
(**a**) Agarose gel electrophoresis for LAMP reaction with varying concentrations of sodium ascorbate (30–60 mM). (**b**) Effect of silver nitrate concentrations (7.5−15 mM) on the color alteration and absorbance of the reaction. N: Negative sample; P: Positive sample. Photos and a graph showing the changes in the absorbance and colorimetric visualization of the reaction mixture measured (**c**) in time-lapse (0–10 min) and (**d**) for 5 min when different temperatures were tested (25–70 °C) for the detection. All experiments were repeated three times. ns: not significant. * *p* < 0.05. *** *p* < 0.001.

Sodium ascorbate acts as a reducing agent during the synthesis of AgNPs. In addition, the antioxidant properties of sodium ascorbate help maintain DNA stability [34]. To evaluate the effectiveness of sodium ascorbate in the LAMP reaction, its concentration was optimized. As illustrated in Figure 8a, the extracted DNA is efficiently amplified in the LAMP reaction at sodium ascorbate concentrations ranging from 30 to 50 mM. However, concentrations exceeding 50 mM adversely affect enzymatic activity, including that of the polymerase, leading to reduced amplification efficiency [35]. Consequently, 50 mM sodium ascorbate was selected as the optimal concentration for colorimetric detection. This concentration balances effective DNA stabilization and minimal impact on enzyme function, ensuring reliable and robust results in the LAMP assay.

The color change was quantitatively analyzed through absorbance measurements. As illustrated in Figure 8b, the absorbance of the positive sample (with the target DNA) is significantly higher, corresponding to a dark yellow color, whereas the negative sample (without the target DNA) shows lower absorbance and a light gray color. Additionally, increasing the concentration of silver nitrate from 7.5 to 15 mM resulted in a noticeable increase in absorbance, indicating a more efficient formation of AgNPs. The reaction required more than 5 min to produce a noticeable color change, as indicated by the markedly increased absorbance compared to that observed when the reaction time was less than 5 min (Figure 8c). Moreover, heat treatment at 65 °C was found to be optimal by comparing the color change and absorbance (Figure 8d). Thus, the conditions for optimal colorimetric detection were established to be 65 °C at 5 min using 15 mM AgNO_3_.

### 3.5. Selectivity and Sensitivity of the One-Pot Colorimetric NAT Platform

**Figure 9:**
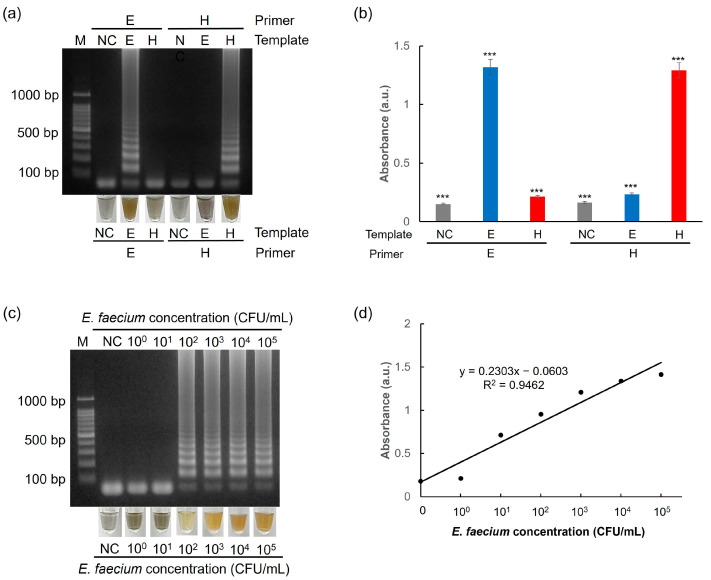
Results showing the selectivity test of (**a**) agarose gel electrophoresis and AgNP-based colorimetry and (**b**) UV–Vis spectrophotometry. Experiments were performed using 10^5^ CFU/mL of *E. faecium*. Results showing the sensitivity test of (**c**) agarose gel electrophoresis and AgNP-based colorimetry and (**d**) a calibration curve between the UV absorbance and *E. faecium* concentration based on AgNP colorimetric assay. All experiments were repeated three times. E: *esp* gene from *E. faecium*. H: *hly*A gene from *L. monocytogenes*. *** *p* < 0.001.

To evaluate the selectivity of the test, two targets were used: the esp gene from *E*. *faecium* and the hlyA gene from *L. monocytogenes*. As shown in Figure 9a,b, *E*. *faecium* target genes were detected only with the esp primer sets, whereas *L*. *monocytogenes* samples were detected only when the *hly*A primer sets were used.

To determine the limit of detection of the one-pot colorimetric NAT platform, GF discs coated with PEI-AgNPs were tested with *E. faecium* at concentrations ranging from 10^0^ to 10^5^ CFU/mL. As illustrated in Figure 9c, DNA extracted from *E. faecium* at concentrations of 10^0^ and 10^1^ CFU/mL is not amplified successfully, indicating that these concentrations are below the detection threshold. In contrast, *E. faecium* DNA is successfully amplified when the target concentration ranges from 10^2^ to 10^5^ CFU/mL. Furthermore, the results of the colorimetric method correspond well with those of electrophoresis, as shown in Figure 9d, where the absorbance measurements provide a detectable signal for *E. faecium* concentrations between 10^2^ and 10^5^ CFU/mL. Therefore, the one-pot colorimetric NAT platform demonstrated a sensitive detection capability, with a limit of detection of approximately 10^2^ CFU/mL.

**Figure 10:**
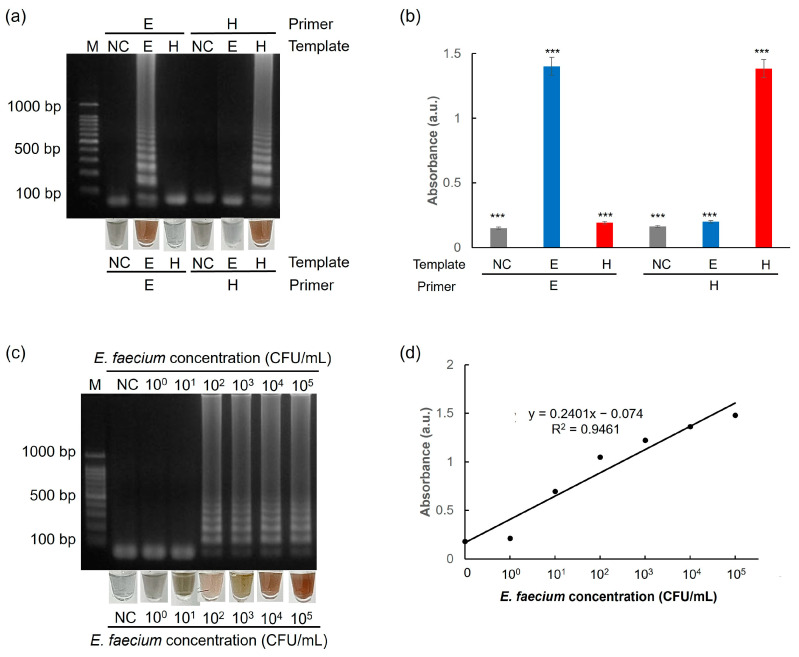
Results showing the selectivity test of (**a**) agarose gel electrophoresis and AgNP-based colorimetry and (**b**) UV–Vis spectrophotometry. Experiments were performed using artificial urine spiked with 10^5^ CFU/mL of *E. faecium*. Results showing the sensitivity test of (**c**) agarose gel electrophoresis and AgNP-based colorimetry and (**d**) a calibration curve between the UV absorbance and *E. faecium* concentration based on AgNP colorimetric assay. Experiments were performed using artificial urine spiked with different concentrations of *E. faecium*. All experiments were repeated three times. E: *esp* gene from *E. faecium*. H: *hly*A gene from *L. monocytogenes*. *** *p* < 0.001.

Moreover, the one-pot colorimetric NAT platform was tested with artificial urine spiked with *E. faecium*. When the *esp* primer sets were used, the platform detected *E. faecium* target genes (Figure 10a,b). Furthermore, *E. faecium* spiked in artificial urine was detected when the target concentration was as low as 10^2^ CFU/mL (Figure 10c,d). These results indicated that our introduced platform could detect targets with high selectivity and sensitivity. These results suggested that the platform can effectively identify targets with high levels of selectivity and sensitivity. A comparative summary of nanoparticle-based DNA extraction methods, including our PEI-AgNP-coated membrane, is presented in Table 2 to highlight the practical advantages of our approach.

## 4. Conclusions

In this study, we developed a NAT platform for seamless operation in molecular diagnostics, which includes nucleic acid extraction and purification, isothermal amplification, and colorimetric detection in one pot. The platform enabled DNA extraction within 10 min. The platform also enabled sample preparation, LAMP reaction, and colorimetric detection within 60 min. Moreover, for both sample extraction and purification, as well as for colorimetric detection, silver ions were used. The system allowed us to isolate and amplify *E. faecium* genes effectively, and the results were verified through on-site visual detection. Rapid and sensitive DNA purification, achieved using a PEI-AgNP-coated GF membrane, supports DNA extraction and purification, considerably simplifying the most time-consuming and labor-intensive processes in molecular diagnostics. In addition, the use of sodium ascorbate, which was used to reduce silver ions to nanoparticles, improved the stability of the LAMP reaction, enabling a clear color difference between the negative and positive samples. The integration of these functionalities into paper discs offers a fast and convenient technique for detecting bacterial DNA that can be effectively applied in field-deployable diagnostic systems.

## Figures and Tables

**Table 1:** Primer sequences used to amplify *E. faecium* and *L. monocytogenes*.

Target Gene	Primer Name	Primer Sequences (5′-3′)
*esp* gene (*Enterococcus faecium*)	F3	CCAGAACACTTATGGAACAG
	B3	GTTGGGCTTTGTGACCTG
	FIP	CGTGTCTCCGCTCTCTTCTTTTTATTT-GCAAGATATTGATGGTG
	BIP	ATCGGGAAACCTGAATTAGAAGAA-GAACTCGTGGATGAATACTTTC
	LB	TGATGTTGACACAACAGTTAAGGG
*hly*A gene (*Listeria monocytogenes*)	F3	TTGCGCAACAAACTGAAGC
	B3	GCTTTTACGAGAGCACCTGG
	FIP	CGTGTTTCTTTTCGATTGGCGTCTTTTTTTCATCCATGGCACCACC
	BIP	CCACGGAGATGCAGTGACAAATGTTTTGGATTTCTTCTTTTTCTCCACAAC
	LB	GCCAAGAAAAGGTTACAAAGATGG

**Table 2:** Comparison of the nanoparticle-based DNA extraction methods.

Method	Advantages	Disadvantages	Reference
Magnetic nanoparticles	- High binding efficiency	- Use of magnets	[36]
	- Easy magnetic separation	- Higher cost	
Gold nanoparticles (AuNPs)	- Tunable surface	- Expensive	[37]
	- High sensitivity	- Complex synthesis	
Silica-coated nanoparticles	- High DNA purity	- Time consuming	[38]
	- Wide applicability	- Relatively expensive	
Carbon nanotubes (CNTs)	- High surface area	- Difficult for purification	[39]
	- Strong adsorption	- Potential for cytotoxicity	
PEI-AgNP-coated GF membrane	- Simple, low-cost, instrument-free	- Limited binding capacity	This study
	- Fast extraction		

## Data Availability

The data presented in this study are available from the corresponding author upon request.
